# A case of unexpected impaired oxygenation due to intraoperative pneumothorax: an adverse event associated with respiratory management with spontaneous respiration in a patient with esophagobronchial fistulae

**DOI:** 10.1186/s40981-017-0102-9

**Published:** 2017-05-30

**Authors:** Seiji Ishikawa, Tsubasa Akune, Tomoko Ishibashi, Koshi Makita

**Affiliations:** 1 Department of Anesthesiology, Graduate School of Medical and Dental Sciences, Tokyo Medical and Dental University, 1-5-45, Yushima, Bunkyo-ku, Tokyo, 113-8519 Japan; 2 Current address: Department of Anesthesiology, Graduate School of Medicine, Juntendo University, 2-1-1, Hongo, Bunkyo-ku, Tokyo, 113-0033 Japan

**Keywords:** Esophagobronchial fistula, Spontaneous respiration, Pneumothorax, Esophageal bypass surgery, Complication, Oxygenation impairment

## Abstract

**Background:**

Respiratory management in patients with esophagobronchial fistulae is challenging since positive pressure ventilation (PPV) may not be feasible due to air leaks and possible risks for regurgitation and aspiration of gastric contents. We and others have previously reported that spontaneous respiration may be one of the good options of respiratory management during general anesthesia in those patients. However, adverse events associated with this respiratory strategy have not been reported previously. We experienced a 77-year-old male patient who suffered unexpected impairment of oxygenation due to intraoperative pneumothorax, which was assumed to have been exacerbated by spontaneous respiration during esophageal bypass surgery.

**Case presentation:**

The patient was planned to undergo esophageal bypass surgery for esophagobronchial fistulae associated with malignant esophageal cancer. Both of two esophagobronchial fistulae were located in the proximal part of the left main bronchus. For the risks of air leaks and aspiration associated with PPV and further damage to the tissue around the fistulae, we decided to maintain spontaneous respiration under general anesthesia and obtain abdominal muscle relaxation with epidural anesthesia. After catheterization of epidural anesthesia, the patient was sedated with 35 mg of intravenous pethidine and was nasotracheally intubated under bronchoscopic guidance. We confirmed that the tip of the tracheal tube was located above the carina. Then anesthesia was induced and maintained with sevoflurane so that his spontaneous respiration could be maintained thereafter. His spontaneous respiration was assisted with 3 cmH_2_O of pressure support. Approximately 60 min into the surgery, percutaneous arterial oxygen saturation (SpO_2_) suddenly dropped from 99 to 89% with an inspiratory fraction of oxygen of 0.4. We assumed that lung atelectasis associated with airway secretion or pulmonary soiling was the most likely reason for impaired oxygenation; however, arterial oxygenation only partially regained even after they were suctioned. After the completion of the surgery, chest X-ray revealed right pneumothorax. After a chest drainage tube was inserted, right pneumothorax was ameliorated and SpO_2_ returned to the baseline level.

**Conclusions:**

Although spontaneous respiration may be useful in a patient with esophagobronchial fistulae, oxygenation can be impaired more seriously than PPV in case intraoperative pneumothorax occurs.

## Background

Respiratory management can be complicated during general anesthesia in patients with esophagobronchial or esophagotracheal fistulae because positive pressure ventilation (PPV) may not be feasible, depending on the size and location of the fistulae, and the risk of regurgitation and aspiration of gastric content may be increased. In these cases, maintaining spontaneous respiration may be useful as a respiratory strategy. In fact, we previously published a case report in which spontaneous respiration was maintained and abdominal muscle relaxation was obtained with epidural anesthesia in a patient who underwent a two-stage surgical procedure for refractory tracheogastric tube fistula [1]. Another case report supported the utility of spontaneous respiration in a patient with tracheoesophageal fistula for whom spontaneous respiration was maintained until veno-venous extracorporeal membrane oxygenation was established [2]. However, adverse events associated with this respiratory strategy have not been reported previously. Here, we report the case of a patient with esophagobronchial fistulae who underwent esophageal bypass surgery with spontaneous respiration and suffered unexpected impairment of oxygenation due to intraoperative pneumothorax, which was assumed to have been exacerbated by spontaneous respiration during surgery.

## Case presentation

Esophageal bypass surgery with gastric tube was planned for a 77-year-old man with esophagobronchial fistulae. He had undergone chemotherapy and radiotherapy for esophageal cancer with possible invasions to the descending aorta and left main bronchus at another hospital, resulting in the formation of fistulae. Since clip plication was unsuccessful and the fistulae remained, he was referred to our hospital for surgical treatment.

**Fig. 1:**
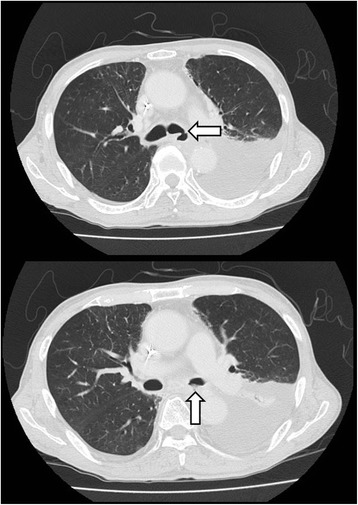
Preoperative computed tomography revealed two esophagobronchial fistulae (*arrows*) at the left main bronchus and pleural effusion in the left thoracic cavity

The patient had a past history of hypertension, diabetes mellitus, bladder cancer, and cerebral infarction. Preoperative arterial blood gas analysis showed a partial oxygen tension (PaO_2_) of 80.5 mmHg, partial carbon dioxide tension (PaCO_2_) of 38.8 mmHg, and pH of 7.44. Preoperative computed tomography showed two esophagobronchial fistulae, with diameters of 7 and 3 mm, located in the proximal part of the left main bronchus, and also demonstrated pleural effusion in the left thoracic cavity (Fig. 1). No bullae were evident in the lungs.

After the patient entered the operating room, an epidural catheter was inserted at the eighth and ninth thoracic interspace. Because no endoscopic images were available, we confirmed the exact location of the esophagobronchial fistulae at the proximal part of the left main bronchus using bronchoscopy after intravenously administering 35 mg of pethidine. Tracheal intubation was performed nasally under bronchoscopic guidance using a long spiral tube with a short cuff (6.0 mm internal diameter, 400 mm length; Phycon, Tokyo, Japan) to a depth of 26 cm. The tip of the tracheal tube was confirmed to be above the carina on bronchoscopy. One-lung ventilation with this tube was planned as an emergency backup by advancing the tube into the right main bronchus in case spontaneous respiration ceased. We chose respiratory management with spontaneous respiration rather than one-lung ventilation in this case since we believed that further damage to the tissue around the fistulae should be avoided. Thus, we decided not to attempt to insert a double-lumen tube into the right main bronchus as a first-line therapy but only for backup plan. We assumed that the tissue around the fistulae may be very fragile after radiotherapy. We also had a jet ventilator and bronchoscope on hand in case hypoxemia developed during one-lung ventilation, so that high-frequency jet ventilation could be performed selectively to the distal part of the left main bronchus beyond the esophagobronchial fistulae. Cardiac surgeons were ready to apply a veno-venous extracorporeal membrane oxygenator in case of severe pulmonary insufficiency as one of the backup plans. After tracheal intubation, anesthesia was induced with 4% sevoflurane and maintained with 1.5–2% sevoflurane. Spontaneous respiration was maintained and was assisted with 3 cmH_2_O of pressure support. Tidal volume and respiratory rate were around 250–400 ml and 10–20 breaths per minute throughout surgery, respectively. Before the abdominal incision was made, 6 ml of 1% lidocaine was injected epidurally, followed by continuous infusion of 0.3% ropivacaine at a rate of 4–6 ml/h to obtain abdominal muscle relaxation. Arterial blood gas analysis showed a PaO_2_ of 252.4 mmHg, a PaCO_2_ of 36.9 mmHg, and a pH of 7.424 with an inspiratory fraction of oxygen (FiO_2_) of 0.6. With an FiO_2_ of 0.4, percutaneous arterial oxygen saturation (SpO_2_) was maintained above 98% during the former part of the surgical procedure.

**Fig. 2:**
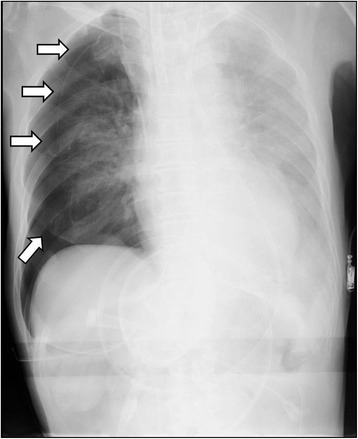
Postoperative chest X-ray showed right pneumothorax (*arrows*). The decrease in radiolucency in the left chest is considered to be due to pleural effusion

Approximately 60 min into the surgery, SpO_2_ suddenly dropped from 98 to 89% with an FiO_2_ of 0.4. We assumed that lung atelectasis associated with airway secretion or pulmonary soiling through the fistulae was the most likely reason for the impaired oxygenation. Bronchofiberscopic examination showed obstruction of the orifice of the left main bronchus with secretion. This secretion was suctioned, and FiO_2_ was increased to 0.6, and pressure support level was increased to 5 cmH_2_O. However, oxygenation only improved partially and arterial blood gas analysis showed a PaO_2_ of 101.7 mmHg with an FiO_2_ of 0.6. After the completion of surgery, chest X-ray revealed right pneumothorax (Fig. 2). After a chest drainage tube was inserted, right pneumothorax was ameliorated, and then SpO_2_ returned to the baseline (99%) with an FiO_2_ of 0.4. The patient was extubated uneventfully in the operating room. The postoperative course was mostly uneventful, except that 650 ml of pleural effusion in the left thoracic cavity was aspirated on postoperative day (POD) 1. The chest tube in the right thoracic cavity was withdrawn on POD 1, and the patient was discharged from hospital on POD 18.

### Discussion

Our management plan, comprising combination of general and epidural anesthesia and maintenance of spontaneous respiration during the surgical procedure, according to our previous report [1], went relatively well except for the unexpected hypoxemia due to intraoperative pneumothorax. The collapse of the right lung may have been facilitated by the spontaneous respiration strategy resulting in impaired oxygenation. Since no bullae were evident on preoperative computed tomography, spontaneous pneumothorax was considered unlikely to have occurred. We believe that this represents the first case report to describe an adverse event associated with maintaining spontaneous respiration in a case of esophagobronchial fistulae.

Optimal respiratory management may differ depending on the site and size of the fistulae between the digestive and respiratory tracts. For example, PPV may be possible with a tracheal fistula with sufficient distance between the fistula and carina, because a tube cuff can easily block the fistula [3]. In a patient with a giant fistula at the carina, one-lung ventilation with a small tracheal tube [4], independent ventilation of each lung with two tracheal tubes [4], or high-frequency jet ventilation [5] may be useful. A veno-venous extracorporeal membrane oxygenator can also be used to improve oxygenation in patients with a fistula between the respiratory and digestive tracts [2]. In this case, we chose respiratory management with spontaneous respiration since we believed that tissues close to the esophagobronchial fistula should not be damaged. Thus, we decided not to attempt to insert a double-lumen tube into the right main bronchus as a first-line therapy but only for backup plan. We assumed that the tissue around the fistulae may be very fragile after radiotherapy. If the fistula had been located in the distal part of the left main bronchus, far from the carina, we may have chosen using a right-sided double-lumen tube.

Maintaining spontaneous respiration is one of the methods of respiratory management chosen in patients with esophagobronchial fistulae during general anesthesia. In the present case, arterial oxygenation and gas exchange were well maintained during the first 60 min with spontaneous respiration. We assume that right pneumothorax occurred during the surgical procedure when SpO_2_ suddenly dropped from 98 to 89%. The collapse of the right lung may have been facilitated with the occurrence of pneumothorax due to spontaneous respiration, while the atelectasis may have been less severe if the lungs had been ventilated with PPV. One of the lessons we learned from this case was that while spontaneous respiration may be useful in a patient with esophagobronchial fistulae, patients may be at higher risk of impaired oxygenation in the event of injury to the parietal pleura during surgery.

Although the exact mechanisms underlying the pneumothorax that occurred in this case during esophageal bypass surgery have not been proven, we assume that the parietal pleura was injured while surgical manipulations were being performed close to the thoracic cavity. Before the present case, our university hospital had treated 48 cases of esophageal bypass surgery and postoperative pneumothorax was encountered in only one case (2.1%) (personal communication with Dr. Nakajima, Department of Surgery of Tokyo Medical and Dental University). Taking the fact that pneumothorax can occur (albeit rarely) into account, spontaneous respiration may have needed to be converted to PPV as soon as feasible after closing both the proximal and distal stumps of the esophagus. Having another backup plan to convert spontaneous respiration to PPV on suspicion of pneumothorax may be important for patients undergoing non-thoracic surgery being performed close to the thoracic cavity. We assume that one-lung ventilation rather than spontaneous respiration should be chosen in hospitals with higher incidence of pneumothorax after esophageal bypass surgery [6].

## Conclusions

In conclusion, although previous reports have shown that spontaneous respiration may be useful in patients with esophagobronchial fistulae, oxygenation can be impaired more seriously than PPV and spontaneous respiration may need to be converted to PPV in case intraoperative pneumothorax arises.

## Abbreviations

FiO_2_: Inspiratory fraction of oxygen
PaCO_2_: Partial carbon dioxide tension
PaO_2_: Partial oxygen tension
POD: Postoperative day
PPV: Positive pressure ventilation
SpO_2_: Percutaneous arterial oxygen saturation

## References

[1] Ishibashi T, Ishikawa S, Suzuki A, Miyawaki Y, Kawano T, Makita K (2016). Successful anesthesia management for 2-stage surgical procedure of a refractory tracheogastric tube fistula after esophagectomy. A A Case Rep.

[2] Collins NF, Ellard L, Licari E, Beasley E, Seevanayagam S, Doolan L (2014). Veno-venous extracorporeal membrane oxygenation and apneic oxygenation for trachea-oesophageal fistula repair in a previously pneumonectomised patient. Anaesth Intensive Care.

[3] Yasuda T, Sugimura K, Yamasaki M (2012). Ten cases of gastro-tracheobronchial fistula: a serious complication after esophagectomy and reconstruction using posterior mediastinal gastric tube. Dis Esophagus.

[4] Roy JS, Girard F, Boudreault D, Pinard AM, Ferraro P (2001). The anesthetic management of a case of tracheogastric fistula. Anesth Analg.

[5] Tsui SL, Lee TW, Chan AS, Lo JR (1991). High-frequency jet ventilation in the anesthetic management of a patient with tracheoesophageal fistula complicating carcinoma of the esophagus. Anesth Analg.

[6] Ananthakrishnan N, Subbarao KS, Parthasarathy G, Kate V, Kalayarasan R (2014). Long term results of esophageal bypass for corrosive strictures without esophageal resection using a modified left colon esophagocoloplasty—a report of 105 consecutive patients from a single unit over 30 years. Hepatogastroenterology.

